# Supplementary material for: Heat adaptation of phage T7 under an extended genetic code
Source: Virus Evol. 2021 Dec 1;7(2):veab100. doi: 10.1093/ve/veab100 (PMC8923235; doi:10.1093/ve/veab100)
Supplement: veab100_Supp [file veab100_supp.zip › Sequencing_Analysis.zip!/Sequencing_Analysis/S2/evidence/RA_15.html]

BRESEQ :: Evidence


| Read alignment evidence... | | | | | | | | | | | |
| --- | --- | --- | --- | --- | --- | --- | --- | --- | --- | --- | --- |
|  | seq id | position | | ref | new | freq | score (cons/poly) | reads | annotation | genes | product |
| \* | NC\_001604.1 | 160 | 0 | T | G | 26.5% | 4673.1 / inf | 4467 | intergenic (–/–) | *–/–* | –/– |
| Reads supporting (aligned to +/- strand):  **ref** base T (1906/1351);  **new** base G (661/513);  **total** (2586/1881) | | | | | | | | | | | |
| Fisher's exact test for biased strand distribution *p*-value = 1.90e-01 | | | | | | | | | | | |
| Kolmogorov-Smirnov test that lower quality scores support variant *p*-value = 1.00e+00 | | | | | | | | | | | |

Only 100 of 4469 total aligned reads displayed.  

`‑‑‑‑‑‑‑‑‑‑‑‑‑TCTCACAGTGTACGGACCTAAAGTTCCCCCATAGGGGGTACCTAAAGCCCAGCCAATCACCTAAAGTCAACCTTCGGTTGACCTTGAGGGTT‑CCCTAAGGGTTGGGGATGACCCTTGGGTTTGTCTTTGGGTGTTACCTTGAGTGTCTCTCTGTGT‑CCCTATCTGTTACAGTCTCCTAAAGTATCCTCCTAAAGTCACCTCCTAACGTCCATCCTAAAGCCAACACCTAAAGCCTACACCTAAAGACCCATCAAGTCAACGCCTATCTTAAAGTTTAAACATAAAGACCAGACCTAAAGACCAGACCTAAAGACACTACATAAAGACCAGACCTAAAGACGCCTTGTTGTTAGCCATAAAGTGATAACCTTTAATCATTGTCTTTATTAATACAACTCACTATAAGGAGAGACAACTTAAAGAGACTTAAAAGATTAATTTAAAATTTATCAAAAAGAGTATTGACTTAAAGTCTAACC  >  NC_001604.1/1‑479`  

                                                                                                                                                                              `|`                                                                                                                                                                                                                                                                                                                                 
`gagatgtgtataagagacaggtgtACGGACCTAAAGTTCCCCCATAGGGGGTACCTAAAGCCCAGCCAATCACCTAAAGTCAACCTTCGGTTGACCTTGAGGGTT‑CCCTAAGGGTTGGGGATGACCCTTGGGTTTGTCTTTGGGTGTTACCTTGAGTGTCTCTCTGTGT‑CCCGATCTGTTACAGTCTCCTAAAGTATCCTCCTAAAGTCACCTCCTAACGTCCATCCTAAAGCCAACACCTAAAGCCTACACCTAAAGACACATCAAGTCAACGCCTATCTTAAAGTTTAAACAtaaagac                                                                                                                                                                                                 <  1:591164/281‑1 (MQ=255)`  
      `ggtataagagacaggtgtACGGACCTAAAGTTCCCCCATAGGGGGTACCTAAAGCCCAGCCAATCACCTAAAGTCAACCTTCGGTTGACCTTGAGGGTT‑CCCTAAGGGTTGGGGATGACCCTTGGGTTTGTCTTTGGGTGTTACCTTGAGTGTCTCACTGTGT‑CCCTATCTGTTACAGTCTCCTAAAGTATCCTCCTAAAGTCACCTCCTAACGTCCATCCTAAAGCCAACACCTAAAGCCTACACCTAAAGACCCATCAAGTCAACGCCTATCTTAAAGTTTAAACAtaaagaccagacc                                                                                                                                                                                           <  1:249983/287‑1 (MQ=255)`  
          `tataagagacaggtACGGACCTAAAGTTCCCCCATAGGGGGTACCTAAAGCCCAGCCAGTCACCTAAAGTCAACCTTCGGTTGACCTTGAGGGTT‑CCCTAAGGGTTGGGGATGACCCTTGGGTTTGTCTTTGGGTGTTACCTTGAGTGTCTCTCTGTGT‑CCCTATCTGTTACAGTCTCCT‑AAGTATCCTCCTAAAGTCACCTCCTAACGTCCATCCTAAAGCCAACACCTAAAGCCTACACCTAAAGACCCATCAAGTCAACGCCTATCTTAAAGTTTAAACAtaaagaccagacctaaag                                                                                                                                                                                      <  1:15636/289‑1 (MQ=255)`  
           `tgtgtataagagacaggACCTAAAGTTCCCCCATAGGGGGTACCTAAAGCCCAGCCAATCACCTAAAGTCAACCTTCGGTTGACCTTGAGGGTT‑CCCTAAGGGTTGGGGATGACCCTTGGGTTTGTCTTTGGGTGTTACCTTGAGTGTCTCTCTGTGT‑CCCTATCTGTTCCAGTCTCCTAAAGTATCCTCCTAAAGTCACCTCCTAACGTCCATCCTAAAGCCAACACCTAAAGCCTACACCTAAAGACCCATCAAGTCAACGCCTATCTTAAAGTTTAAACAtaaagaccagacctaaag                                                                                                                                                                                      <  1:824577/286‑1 (MQ=255)`  
           `aagagacaggtgtACGGACCTAAAGTTCCCCCATAGGGGGTACCTAAAGCCCAGCCAATCACCTAAAGTCAACCTTCGGTTGACCTTGAGTGTT‑CCCTAAGGGTTGGGGATGACCCTTGGGTTTGTCTTTGGGTGTTACCTTGAGTGTCTCTCTGTGT‑CCCTATCTGTTACAGTCTCCTAAAGTATCCTCCTAAAGTCACCTCCTAACGTCCATCCTAAAGCCAACACCTAAAGCCTACACCTAAAGACCCATCAAGTCAACGCCTATCTTAAAGTTTAAACAtaaagaccagacctaaag                                                                                                                                                                                      <  1:71820/292‑1 (MQ=255)`  
             `gctcggagatgtgtataagagacagcccccATAGGGGGTACCTAAAGCCCCGCCAATCACCTAAAGTCAACCTTCTGTTGACCTTGAGGGTT‑CCCTAAGGGTTGGGGATGACCCTTGGGTTTGTCTTTGGGTGTTACCTTGAGTGTCTCTCTGTGT‑CCCGATCTGTTACAGTCTCCTAAAGTATCCTCCTAAAGTCACCTCCTAACGTCCATCCTAAAGCCAACACCTAAAGCCTACACCTAAAGACCCATCAAGTCAACGCCTATCTTAAAGTTTAAACAtaaagaccagacctaaagac                                                                                                                                                                                    <  1:784061/276‑1 (MQ=25)`  
                    `gtgtACGGACCTAAAGTTCCCCCATAGTGGGTACCTAAAGCCCAGCCAATCACCTAAAGTCAACCTTCGGTTGACCTTGAGGGTT‑CCCTAAGGGTTGGGGATGACCCTTGGGTTTGTCTTTGGGTGTTACCTTGAGTGTCTCTCTGTGT‑CCCTATCTGTTACAGTCTCCTAAAGTATCCTCCTAAAGTCACCTCCTAACGTCCATCCTAAAGCCAACACCTAAAGCCTACACCTAAAGACCCATCAAGTCAACGCCTATCTTAAAGTTTAAACAtaaagaccagacctaaagaccagaccc                                                                                                                                                                             >  1:146754/1‑300 (MQ=255)`  
                    `gtgtACGGACCTAAAGTTCCCCCATAGGGGGTACCTAAAGCCCAGCCAATCACCTAAAGTCAACCTTCGGTTGACCTTGAGGGTT‑CCCTAAGGGTTGGGGATGACCCTTGGGTTTGTCTTTGGGTGTTACCTTGAGTGTCTCTCTGTGT‑CCCTATCTGTTCCAGTCTCCTAAAGTATCCTCCTAAAGTCACCTCCTAACGTCCATCCTAAAGCCAACACCTAAAGCCTACACCTAAAGACCCATCAAGTCAACGCCTATCTTAAAGTTTAAACAtaaagaccagacctaaagctgtctctt                                                                                                                                                                             >  1:583536/1‑292 (MQ=255)`  
                    `gtgtACGGACCTAAAGTTCCCCCATAGGGGGTACCTAAAGCCCAGCCAATCACCTAAAGTCAACCTTCGGTTGACCTTGAGGGTT‑CCCTAAGGGTTGGGGATGACCCTTGGGTTTGTCTTTGGGTGTTACCTTGAGTGTCTCTCTGTGT‑CCCTATCTGTTACAGTCTCCTAAAGTATCCTCCTAAAGTCACCTCCTAACGTCCATCCTAAAGCCAACACCTAAAGCCTACACCTAAAGACCCATCAAGTCAACGCCTATCTTAAAGTTTAAACAtaaagacctgtctcttatacacatctc                                                                                                                                                                             >  1:478902/1‑282 (MQ=255)`  
                    `gtgtACGGACCTAAAGTTCCCCCATAGGGGGTACCTAAAGCCCAGCCAATCACCTAAAGTCAACCTTCGGTTGACCTTGAGGGTT‑CCCTAAGGGTTGGGGATGACCCTTGGGTTTGTCTTTGGGTGTTACCTTGAGTGTCTCTCTGTGT‑CCCTATCTGTTACAGTCTCCTAAAGTATCCTCCTAAAGTCACCTCCTAACGTCCATCCTAAAGCCAACACCTAAAGCCTACACCTAAAGACCCATCAAGTCAACGCCTATCTTAAAGTTTAAACATaaagacccgacctaaagaccagacct                                                                                                                                                                             >  1:526782/1‑301 (MQ=255)`  
                    `gtgtACGGACCTAAAGTTCCCCCATAGGGGGTACCTAAAGCCCAGCCAATCACCTAAAGTCAACCTTCGGTTGACCTTGAGGGTT‑CCCTAAGGGTTGGGGATGACCCTTGGGTTTGTCTTTGGGTGTTACCTTGAGTGTCTCTCTGTGT‑CCCTATCTGTTACAGTCTCCTAAAGTATCCTCCTAAAGTCACCTCCTAACGTCCATCCTAAAGCCAACACCTAAAGCCTACACCTAAAGACCCATCAAGTCAACGCCTATCTTAAAGTTTAAACATaaagaccagacctaaagaccagacct                                                                                                                                                                             >  1:643708/1‑301 (MQ=255)`  
                    `gtgtACGGACCTAAAGTTCCCCCATAGGGGGTACCTAAAGCCCAGCCAATCACCTAAAGTCAACCTTCGGTTGACCTTGAGGGTT‑CCCTAAGGGTTGGGGATGACCCTTGGGTTTGTCTTTGGGTGTTACCTTGAGTGTCTCTCTGTGT‑CCCTATCTGTTACAGTCTCCTAAAGTATCCTCCTAAAGTCACCTCCTAACGTCCATCCTAAAGCCAACACCTAAAGACTACACCTAACGACCCATCAAGTCAACGCCTATCTTAAAGTTTAAACATaaagaccagacctaaagaccagacct                                                                                                                                                                             >  1:284304/1‑301 (MQ=255)`  
                    `gtgtACGGACCTAAAGTTCCCCCATAGGGGGTACCTAAAGCCCAGCCAATCACCTAAAGTCAACCTTCGGTTGACCTTGAGGGTT‑CCCTAAGGGTTGGGGATGACCCTTGGGTTTGTCTTTGGGTGTTACCTTGAGTGTCTCTCTGTGT‑CCCTATCTGTTACAGACTCCTAAAGTATCCTCCTAAAGTCACCTCCTAACGTCCATCCTAAAGCCAACACCTAAAGCCTACACCTAAAGACCCATCAAGTCAACGCCTATCTTAAAGTTTAAACATaaagaccagacctaaataccagacct                                                                                                                                                                             >  1:323254/1‑301 (MQ=255)`  
                    `gtgtACGGACCTAAAGTTCCCCCATAGGGGGTACCTAAAGCCCAGCCAATCACCTAAAGTCAACCTTCGGTTGACCTTGAGGGTT‑CCCTAAGGGTTGGGGATGACCCTTGGGTTTGTCTTTGGGTGTTACCTTGAGTGTCTCTCTGTGT‑CCCGATCTGTTACAGTCTCCTAAAGTATCCTCCTAAAGTCACCTCCTAACGTCCATCCTAAAGCCAACACCTAAAGCCTACACCTAAAGACTCATCAAGTCAACGCCTATCTTAAAGTTTAAACATaaagaccagacctaaagaccatacct                                                                                                                                                                             >  1:733571/1‑301 (MQ=255)`  
                    `gtgtACGGACCTAAAGTTCCCCCATAGGGGGTACCTAAAGCCCAGCCAATCACCTAAAGTCAACCTTCGGTTGACCTTGAGGGTT‑CCCTAAGGGTTGGGGATGACCCTTGGGTTTGTCTTTGGGTGTTACCTTGAGTGTCTCTCTGTGT‑CCCGATCTGTTACAGTCTCCTAAAGTATCCTCCTAAAGTCACCTCCTAACGTCCATCCTAAAGCCAACACCTAAAGCCTACACCTAAAGACCCATCAAGTCAACGCCTATCTTAAAGTTTAAACAtaaagaccctgtctcttatacacatct                                                                                                                                                                             >  1:424061/1‑282 (MQ=255)`  
                    `gtgtACGGACCTAAAGTTCCCCCATAGGGGGTACCTAAAGCCCAGCCAATCACCTAAAGTCAACCTTCGGTTGACCTTGAGGGTT‑CCCTAAGGGTTGGGGATGACCCTTGGGTTTGTCTTTGGGTGTTACCTTGAGTATCTCTCTGTGT‑CCCTATCTGTTACAGTCTCCTAAAGTATCCTCCTAAAGTCACCTCCTAACGTCCATCCTAAAGCCAACACCTAAAGCCTACACCTAAAGACCCATCAAGTCAACGCCTATCTTAAAGTTTAAACATaaagaccagacctaaagaccagacct                                                                                                                                                                             >  1:343103/1‑301 (MQ=255)`  
                    `gtgtACGGACCTAAAGTTCCCCAATAGGGGGTACCTAAAGCCCAGCCAATCACATAAAGTCAAACTTCGGTTGACCTTGAGGGTT‑CCCTAAGGGTTAGGGATGACCCTTGGGTTTGTCTTTGGGTGTTACCTTGAGTGTCGCTCTGTGT‑CCCTATCTGTTACAGTCTCTTAAAGTATCCTCGTAAGGTATCTTACTAACGTCCATCATAACGCTAACAACTAAAGACTACAACCAAAGAACCATCAAGTAAAACAAAAAATTAAGGGTTAAAAAtaaagacaaaccctaaagcaaagacat                                                                                                                                                                             >  1:84300/1‑292 (MQ=17)`  
                      `gtACGGACCTAAAGTTCCCCCATAGGGGGTACCTAAAGCCCAGCCCATCACCTAAAGTCAACCTTCGGTTGACCTTGAGGGTT‑CCCTAAGGGTTGGGGATGACCCTTGGGTTTGTCTTTGGGTGTTACCTTGCGTGTCTCTCTGTGT‑CCCTATCTGTTACAGTCTACTACAGTATCCTCATAAAGTCACCTCCTAACGTCCATCCTAAAGCCAACACCTAAAGCCTACACCTAAAGATCCATCAAGTCAACACCTATCTTAAAGTTTAAATATACataccacacctacagaccagacctaa                                                                                                                                                                           >  1:501719/1‑301 (MQ=25)`  
                      `gtACGGACCTAAAGTTCCCCCATAGGGGGTACCTAAAGCCCAGCCAATCACCTAAAGTCAACCTTCGGTTGACCTTGAGGGTT‑CCCTAAGGGTTGGGGATGACCCTTGGGTTTGTCTTTGGGTGTTACCTTGAGTGTCTCTCTGTGT‑CCCTATCTGTTACAGTCTCCTAAAGTATCCTCCTAAAGTCACCTCCTAACGTCCATCCTAAAGCCAACACCTAAAGCCTACACCTAAAGACCCATCAAGTCAACGCCTATCTTAAAGTTTAAACATAAagaccagacctaaagaccagacctaa                                                                                                                                                                           >  1:798600/1‑301 (MQ=255)`  
                      `gtACGGACCTAAAGTTCCCCCATAGGGGGTACCTAAAGCCCAGCCAATCACCTAAAGTCAACCTTCGGTTGACCTTGAGGGTT‑CCCTAAGGGTTGGGGATGACCCTTGGGTTTGTCTTTGGGTGTTACCTTGAGTGTCTCTCTGTGT‑‑CCTATCTGTTACAGTCTCCTAAAGTATCCTCCTAAAGTCACCTCCTAACGTCCATCCTAAAGCCAACACCTAAAGCCTACACCTAAAGACCCATCAAGTCAACGCCTATCTTAAAGTTTAAACATAAAgaccagacctaaagaccagacctaaa                                                                                                                                                                          >  1:121184/1‑301 (MQ=255)`  
                      `gtACGGACCTAAAGTTCCCCCATAGGGGGTACCTAAAGCCCAGCCAATCACCTAAAGTCAACCTTCGGTTGACCTTGAGGGTT‑CCCTAAGGGTTGGGGATGACCATTGGGTTTGTCTTTGGGTGTTACCTTGAGTGTCTCTCTGTGT‑CCCTATCTGTTACAGTCTCCTAAAGTATCCTCCTAAAGTCACCTCCTAACGTCCATCATAAAGCCAACACCTAAAGCCTACACCTAAAGACCCATCAAGTCAACGCCTATCTTAAAGTTTAAACATAAagaccagacctaaagaccagacctaa                                                                                                                                                                           >  1:576367/1‑301 (MQ=255)`  
                      `gtACGGACCTAAAGTTCCCCCATAGGGGGTACCTAAAGCCCAGCCAATCACCTAAAGTCAACCTTCGGTTGACCTTGAGGGTT‑CCCTAAGGGCTGGGGATGACCCTTGGGTTTGTCTTTGGGTGTTACCTTGAGTGTCTCTCTGTGT‑‑CCTATCTGTTACAGTCTCCTAAAGTATCCTCCTAAAGTCACCTCCTAACGTCCATCCTAAAGCCAACACCTAAAGCCTACACCTAAAGACCCATCAAGTCAACGCCTATCTTAAAGTTTAAACATAAAgaccagacctaaagaccagacctaaa                                                                                                                                                                          >  1:551203/1‑301 (MQ=255)`  
                        `caggACCTAAAGTTCCCCCATAGGGGGTACCTAAAGCCCAGCCAATCACCTAAAGTCAACCTTCGGTTTTCCTTGAGGGTT‑CCCTAAGGGTTGGGGATGACCCTTGGGTTTGTCTTTGGGTGTTACCTTGAGTGTCTCTCTGTGT‑CCCTATCTGTTACAGTCTCCTAAAGTATCCTCCTAAAGTCACCTCCTAACGTCCATCCTAAAGCCAACACCTAAAGCCTACACCTAAAGACCCATCAAGTCAACGCCTATCATAAAGTTTAAACATAAAGaccagacctaaagaccagacctaaag                                                                                                                                                                         <  1:31004/299‑1 (MQ=255)`  
                        `aCGGACCTAAAGTTCCCCCATAGGGGGTACCTAAAGCCCAGCCAATCACCTAAAGGCAACCTTCGGTTGACCTTGAGGGTT‑CCCTAAGGGTTGGGGATGACCCTTGGGTTTGTCTTTGGGTGTTACCTTGAGTGTCTCTCTGTGT‑CCCTATCTGTTACAGTCTCCTAAAGTATCCTCCTAAAGTCACCTCCTAACGTCCATCCTAAAGCCAACAACTAAAGCCTACACCTAAAGACCCATCAAGTCAACGCCTATCTTAAAGTTTAAACATAAAGaccagacctaaagaccagacctaaag                                                                                                                                                                         <  1:409935/301‑1 (MQ=255)`  
                          `ggACCTAAAGTTCCCCCATAGGGGGTACCTAAAGCCCAGGCAATCAACTAAAGTCAACCTTCGGTTGACCTTGAGGGTT‑CCCTAAGGGTTGGGGATGACACTTGGGTTTGTCTTTGGGTGTTACCTTGAGTGTCTCTCTGTGT‑CCCTATCTGTTACAGTCTCCTAAAGTATCCTCCTAAAGTCACCTCCTAACGTCCATCCTAAAGCCAACACCTAAAGCCTACACCTAAAGACCCATCAAGTCAACGCCTATCTTAAAGTTTAAACATAAAGACcagacctaaagaccagacctaaagac                                                                                                                                                                       <  1:18643/301‑1 (MQ=255)`  
                          `aagatacaggTTCCCCCATAGGTGGTACCTAAAGCCCAGCCAATCACCTAAAGTCAACCGTCTGTTGACCTTGAGGGTT‑CCCTAAGGGTTGGGGATGACCCTTGGGTTTGTATTTGGGTGTTACCTTGAGTGTCTCTCTGTGT‑CCCGATCTGTTACAGTCTCCTAAAGTATCCTCCTCAAGTCACCTCCTAACGTCCATCCTAAAGCCAACACCTAAAGCCTACACCTAAAGACCCATCAAGGCAACGCCTATCTTAAAGTTTAAACATAAAGACcagacctaaagaccagacctaaagac                                                                                                                                                                       <  1:537640/292‑1 (MQ=255)`  
                           `gACCTAAAGTTCCCCCATAGGGGGTACCTAAAGCCCAGCCAATCACCTAAAGTCAACCTTCGGTTGACCTTGAGGGTT‑CCCTAAGGGTTGGGGATGACCCTTGGGTTTGTCTTTGGGTGTTACCTTGAGTGTCTCTCTGTGT‑CCCTATCTGTTCCAGTCTCCTAAAGTATCCTCCTAAAGTCACCTCCTAACGTCCATCCTAAAGCCAACACCTAAAGCCTACACCTAAAGACCCATCAAGTCAACGCCTATCTTAAAGTTTAAACATAAAGACCAGACCTAAAGACCAGACCTAAAGaca                                                                                                                                                                      >  1:741801/1‑301 (MQ=255)`  
                           `gACCTAAAGTTCCCCCATAGGGGGTACCTAAAGCCCAGCCAATCACCTAAAGTCAACCTTCGGTTGACCTTGAGGGTT‑CCCTAAGGGTTGGGGATGACCCTTGGGTTTGTCTTTGGGTGTTACCTTGAGTGTCTCTCTGTGT‑CCCTATCTGTTCCAGTATCCTAAAGTATCCTCCTAAAGTCACCTCCTAACGTCCATCCTAAAGCCAACACCTAAAGCCTACACCTAAAGACCCATCAAGTCAACGCCTATCTTAAAGTTTAAACATAAAGACCAGACCTAAAGACCAGACCTAAAGaca                                                                                                                                                                      >  1:278816/1‑301 (MQ=255)`  
                           `gACCTAAAGTTCCCCCATAGGGGGTACCTAAAGCCCAGCCAATCACCTAAAGTCAACCTTCGGTTGACCTTGAGGGTT‑CCCTAAGGGTTGGGGATGACCCTTGGGTTTGTCTTTGGGTGTTACCTTGAGTGTCTCTCTGTGT‑CCCTATCTGTTACAGTCTCCTAAAGTATCCTCCTAAAGTCACCTCCTAACGTCCATCCTAAAGCCAACACCTAAAGCCTACACCTAAAGACCCATCAAGTCAACGCCTATCTTAAAGTTTAAACAtaaagaccagacctaaagaccctgtctcttataa                                                                                                                                                                      >  1:61389/1‑288 (MQ=255)`  
                           `gACCTAAAGTTCCCCCATAGGGGGTACCTAAAGCCCAGCCAATCACCTAAAGTCAACCTTCGGTTGACCTTGAGGGTT‑CCCTAAGGGTTGGGGATGACCCTTGGGTTTGTCTTTGGGTGTTACCTTGAGTGTCTCTCTGTGT‑CCCGGTCTGTTACAGTCTCCTAAAGTATCCTCCTAAAGTCACCTCCTAACGTCCATCCTAAAGCCAACACCTAAAGCCTACACCTAAAGACCCATCAAGTCAACGCCTATCTTTAAGTTTAAACATAAAGACCAGACCTAAAGACCAGACCTAAAGaca                                                                                                                                                                      >  1:335129/1‑301 (MQ=255)`  
                           `gACCTAAAGTTCCCCCATAGGGGGTACCTAAAGCCAAGCCAATCACCTAAAGTCAACCTTCGGTTGACCTTGAGGGTT‑CCCTAAGGGTTGGGGATGACCCTTGGGTTTGTCTTTGGGTGTTACCTTGAGTGTCTCTCTGTGT‑CCCTATCTGTTACAGTCTCCTAAAGTATCCTCCTAAAGTCACCTCCTAACGTCCATCCTAAAGCCAACACCTAAAGCCTACACCTAAAGACCCATCAAGTCAACGCCTATCTTAAAGTTTAAACATAAAGACCAGACCTAAAGACCAGACCTAAAGaca                                                                                                                                                                      >  1:241273/1‑301 (MQ=255)`  
                           `aaCCTAAAGTTCCCCCATAGGAGGTACCTAAAGCCCAGCCAATCACGTAAAGTCAACCTTCGGTTTCCCTTGAGGGTT‑CCCTAAGGATTGGGGATGACCCTTGGGTTTATCTTTGGGTGTTACCTTGAGTGTCTCTCTGTGTCCCCTATCTGTTACAGTCTCCTAAAGTATCCGCCTAAAGTAACCTCCTAACGTCCATCCTAAAGCCAACACCTAAAGCCTACACCTAAAGACCCATCAAGTCAACGCCTATCTTAAAGTTTAAACATAAAGACcagacctaaagaccagacctaaagac                                                                                                                                                                       <  1:484752/300‑1 (MQ=37)`  
                            `taagagacagcccccATAGGGGGTACCTAAAGCCCAGCCAATCACCTAAAGTCAACCTTCGGTTGGCCTTGAGGGTT‑CCCTAAGGGTTGGGGATGACCCTTGGGTTTGTCTTTGGGTGTTACCTTGAGTGTCTCTCGGTGT‑CCCTATCTGTTACAGTCTCCTAAAGTATCCTCCTAAAGTCACCTCCTAACGTCCATCCTAAAGCCAACACCTAAAGCCTACACCTAAAGACCCATCAAGTCAACGCCTATCTTAAAGTTTAAACATAAAGACCAGACCTAAAGACCAGACCTAAAGacac                                                                                                                                                                     <  1:291383/291‑1 (MQ=255)`  
                             `ccTAAAGTTCCCCCATAGGGGGTACCTAAAGCCCAGCCAATCACCTAAAGTCAACCTTCGGTTGACCTTGAGGGTT‑CCCTAAGGGTTGGGGATGACCCTTGGGTTTGTCTTTGGGTGTTACCTTGAGTGTCTCTCTGTGT‑CCCGATCTGTTACAGTCTCCTAAAGTATCCTCCTAAAGTCACCTCCTAACGTCCATCCTAAAGCCAACACCTAAAGCCTACACCTACAGACCCATCAAGTCAACGCCTATCTTAAAGTTTAAACATAAAGACCAGACCTAAAGACCAGACCTAAAGACACt                                                                                                                                                                    >  1:178198/1‑301 (MQ=255)`  
                                 `agatgtgtataagagacagaCCTAAAGCCCAGCCAATCACCTAAAGTCAACCTTCGGTTGACCTTGAGGGTT‑CCCTAAGGGTTGGGGATGACCCTTGGGTTTGTCTTTGGGTGTTACCTTGAGTGTCTCTCTGTGT‑CCCGATCTGTTACAGTCTCCTAAAGTATCCTCCTAAAGTCACCTCCTAACGTCCATCCTAAAGCCAACACCTAAAGCCTACACCTAAAGACCCATCAAGTCAACGCCTATCTTAAAGTTTAAACATAAAGACCAGACCTAAAGACCAGACCTAAAGACACTACAt                                                                                                                                                                <  1:381091/282‑1 (MQ=255)`  
                                 `aaGTTCCCCCATAGGGGGTACCTAAAGCCCAGCCAATCACCTAAAGTCAACCTTCGGTTGACCTTGAGGGTT‑CCCTAAGGGTTGGGGATGACCCTTGGGTTTGTCTTTGGGTGTTACCATGAGTGTCTCTCTGTGT‑CCCTATCTGTTACAGTCTCCTAAAGTATCCTCCTAAAGTCACCTCCTAACGTCCATCCTAAAGCCAACACCTAAAGCCTACACCTAAAGACCCATCAAGTCAACGCCTATCTTAAAGTTTAAACATAAAGACcagacctaaagaccagacctaaagacctgtctc                                                                                                                                                                >  1:460272/1‑294 (MQ=255)`  
                                     `gcccccATAGGGGGGACCTAAAGCCCAGCCAATCACCTAAAGTCAACCTTCGGTTGACCTTGGGGGTT‑CCCCAAGGGTTGGGGATGACCCTTGGGTTTGTCTTTGGGTGTTACCTTGAGTGTCTCTCTGTGT‑CCCTATCTGTTCCAGTCTCCTAAAGTATCCTCCTAAAGTCACCTCCTAACGTCCATCCTAAAGCCAACACCTAAAGCCTACACCTAAAGACCCATCAAGTCAACGCCTATCTTAAAGTTTAAACATAAAGACCAGACCTAAAGACCAGACCTAAAGACACTACATAAag                                                                                                                                                            <  1:10821/300‑1 (MQ=255)`  
                                      `cccccATAGGGGGTACCTAAAGCCCAGCCAATCACCTAAAGTCAACCTTCGGTTGACCTTGAGGGTT‑CCCTAAGGGTTGGGGATGACCCTTTGGTTTGTCTTTGGGTGTTACCTTGAGTGTCTCTCTGTGT‑CCCTATCTGTTACAGTCTCCTAAAGTATCCTCCTAAAGTCACCTCCTAACGTCCATCCTAAAGCCAACACCTAAAGCCTACACCTAAAGACCCATCAAGTCAACGCCTATCTTAAAGTTTAAACATAAAGACCAGACCTAAAGACCAGACCTAAAGACATTACacggtct                                                                                                                                                           >  1:817750/1‑295 (MQ=255)`  
                                      `cccccATAGGGGGTACCTAAAGCCCAGCCAATCACCTAAAGTCAACCTTCGGTTGACCTTGAGGGTT‑CCCTAAGGGTTGGGGATGACCCTTGGGTTTGTCTTTGGGTGTTACCTTGAGTGTCTCTCTGTGT‑CCCTATCTGTTACATTCTCCTAAAGTATCCTCCTAAAGTCACCTCCTAACGTCCATCCTAAAGCCAACACCTAAAGCCTACACCTAAAGACCCATCAAGTCAACGCCTATCTTAAAGTTTAAACATAAAGACCAGACCTAAAGACCAGACCTAAAGACACTACATaaaaa                                                                                                                                                           >  1:373652/1‑299 (MQ=255)`  
                                      `cccccATAGGGGGTACCTAAAGCCCAGCCAATCACCTAAAGTCAACCTTCGGTTGACCTTGAGGGTT‑CCCTAAGGGTTGGGGATGACCCTTGGGTTTGTCTTTGGGTGTTACCTTGAGTGTCTCTCTGTGT‑CCCTATCTGTTACAGTCTCCTAAAGTATCCTCCTAAAGTCACCTCCTAACGTCCATCCTAAAGCCAACACCTAAAGCCTACACCTAAAGACCCATCAAGTCAACGCCTATCTTAAAGTTTAAACATAAAGACCAGACCTAAAGACCAGACCTAAAGACACTACATAAaga                                                                                                                                                           <  1:749922/301‑1 (MQ=255)`  
                                       `ccccATAGGGGGTACCTAAAGCCCAGCCAATCACCTAAAGTCAACCTTCGGTTGACCTTGAGGGTT‑CCCTAAGGGTTGGGGATGACCCTTGGGTTTGTCTTTGGGTGTTACCTTGAGTGTCTCTCTGTGT‑CCCTATCTGTTACAGTCTCCTAAAGTATCCTCCTAAAGTCACCTCCTAACGTCCATCCTAAAGCCAACACCTAAAGCCTACACCTAAAGACCCATCAAGTCAACGCCTATCTTAAAGTTTAAACATAAAGACCAGACCTAAAGACCAGACCTAAAGACACTACATAAagac                                                                                                                                                          <  1:403499/301‑1 (MQ=255)`  
                                        `gtataagtgacagccTAAAGCCCAGCCAATCACCTAAAGTCAACCTTCGGTTGGCCTTGAGGGTT‑CCATAAGGGTTGGGGATGACCCTTGGGTTTGTCGTTGGTTGTGAACTGGAGTGTCTCTCTGTGTCCCCTATCTGTCACAGTCTCATAAAGTAGCCTCCTAAAGTCACCTCCTAACGTCCATCCTAAAGCCAACACCTAAAGCCTACACCTAAAGACCCATCAAGTCAACGCCTATCTTAAAGTTTAAACATAAAGACCAGACCTAAATACCAGACCTAAAGACACTACATAAagac                                                                                                                                                          <  1:432/288‑1 (MQ=37)`  
                                        `cccATAGGGGGTACCTAAAGCCCAGCCAATCACCTAAAGGCAACCTTCGGTTGACCTTGAGGGTT‑CCCTAAGGGTTGGGGATGACCCTTGGGTTTGTCTTTGGGTGTTACCTTGAGTGTCTCTCTGTGT‑CCCGATCTGTTACAGTCTCCTAAAGTATCCTCCTAAAGTCACCTCCTAACGTCCATCCTAAAGCCAACACCTAAAGCCTACACCTAAAGACCCATCAAGTCAACGCCTATCTTAAAGTTTAAACATAAAGACCAGACCTAAAGACCAGACCTAAAGACACTACATAAagacc                                                                                                                                                         <  1:810454/301‑1 (MQ=255)`  
                                             `aGGGGGTACCTAAAGCCCAGCCAATCACCTAAAGTCAACCTTCGGTTGACCTTGAGGGTT‑CCCTAAGGGTTGGGGATGACCCTTGGGTTTGTCTTTGGGTGTTACCTTGAGTGTCTCTCTGTGT‑CCCTATCTGTTACAGTCTCCTAAAGTATCCTCCTAAAGTCACCTCCTAACGTCCATCCTAAAGCCAACACCTAAAGCCTACACCTAAAGACCCATCAAGTCAACGCCTATCTTAAAGTTTAAACATAAAGACCAGACCTAAAGACCAGACCTAAAGACACTACATAAagaccagacc                                                                                                                                                    <  1:202263/301‑1 (MQ=255)`  
                                              `gggggTACCTAAAGCCCAGCCAATCACCTAAAGTCAACCTTCGGTTGACCTTGAGGGTT‑CCCTAAGGGTTGGGGATGACCCTTGGGTTTGTCTTTGGGTGTTACCTTGAGTGTCTCTCTGTGT‑CCCGATCTGTTACAGTCTCCTAAAGTATCCTCCTAAAGTCACCTCCTAACGTCCATCCTAAAGCCAACACCTAAAGCCTACACCTAAAGACCCATCAAGTCAACGCCTATCTTAAAGTTTAAACATAAAGACCAGACCTAAAGACCAGACCTAAAGACACTACATAAAGACCAGACCt                                                                                                                                                   >  1:129865/1‑301 (MQ=255)`  
                                                  `gTACCTAAAGCCCAGCCAATCACCTAAAGTCAACCTTCGGTTGACCTTGAGGGTT‑CCCTAAGGGTTGGGGATGACCCTTGGGTTTGTCTTTGGGTGTTACCTTGAGTGTCTCTCTGTGT‑CCCTATCTGTTCCAGTCTCCTAAAGTATCCTCCTAAAGTCACCTCCTAACGTCCATCCTAAAGCCAACACCTAAAGCCTACACCTAAAGACCCATCAAGTCAACGCCTATCTTAAAGTTTAAACATAAAGACCAGACCTAAAGACCAGACCTAAAGACACTACATAAAGACCAGACCTAAAg                                                                                                                                               <  1:194059/301‑1 (MQ=255)`  
                                                    `aCCTAAAGCCCAGCCAATCACCTAAAGTCAACCTTCGGTTGACCTTGAGGGTT‑CCCTAAGGGTTGGGGATGACCCTTGGGTTTGTCTTTGGGTGTTACCTTGAGTGTCTCTCTGTGT‑CCCGATCTGTTACAGTCTCCTAAAGTATCCTCCTAAAGTCACCTCCTAACGTCTATCCTAAAGCCAACACCTAAAGCCTACACCTAAAGACCCATCAAGTCAACGCCTATCTTAAAGTTTAAACATAAAGACCAGACCTAAAGACCAGACCTAAAGACACTACATAAAGACCAGACCTAAAGAc                                                                                                                                             >  1:620802/1‑301 (MQ=255)`  
                                                    `aCCTAAAGCCCAGCCAATCACCTAAAGTCAACCTTCGGTTGACCTTGAGGGTT‑CCCTAAGGGTTGGGGATGACCCTTGGGTTTGTCTTTGGGTGTTACCTTGAGTGTCTCTCTGTGT‑CCCGATCTGTTACAGTCTCCTAAAGTATCCTCCTAAAGTCACCTCCTAACGTCCATCCTAAAGCCAACACCTAAAGCCTACACCTAAAGACCCATCAAGTCAACGCCTATCTTAAAGTTTAAACATAAAGACCAGACCTAAAGACCAGACCTAAAGACACTACATAAAGACCAGACCTAAAGAc                                                                                                                                             >  1:628332/1‑301 (MQ=255)`  
                                                     `ccTAAAGCCCAGCCAATCACCTAAAGTCAACCTTCGGTTGACCTTGAGGGTT‑CCCTAAGGGTTGGGGATGACCCTTGGGTTTGCCTTTGGGTGTTACCTTGAGTGTCTCTCTGTGT‑CCCGATCTGTTACAGTCTCCTAAAGTATCCTCCTCAAGTCACCTCCTCACGCCCATCCTAAAGCCAACACCTAAAGCCTACACCTAAAGACCCATCAACTCAACGCCTCTCTTAAAGTTTAAACATAAAGACCAGACCTAAAGACCAGACCTAAAGACACTAAATAAAGAACAGACCTAAAGAca                                                                                                                                            >  1:352454/1‑300 (MQ=255)`  
                                                      `cTAAAGCCCAGCCAATCACCTAAAGTCAACCTTCGGTTGACCTTGAGGGTTCCCCTAAGGGTTGGGGATGACCCTTGGGTTTGTCTTTGGGTGTTACCTTGAGTGTCTCTCTGTGT‑CCCGATCTGTTACAGTCTCCTAAAGTATCCTCCTAAAGTCACCTCCTAACGTCCATCCTAAAGCCAACACCTAAAGCCTACACCTAAAGACCCATCAAGTCAACGCCTATCTTAAAGTTTAAACATAAAGACCAGACCTAAAGACCAGACCTAAAGACACTACATAAAGACCAGACCTAAAGACg                                                                                                                                            >  1:696889/1‑301 (MQ=255)`  
                                                                `gCCAATCACCTAAAGTCAACCTTCGGTTGACCTTGAGGGTT‑CCCTAAGGGTTGGGGATGACCCTTGGGTTTGTCTTTGGGTGTTACCTTGAGTGTCTCTCTGTGG‑CCCTATCTGTTCCAGTCTCCTAAAGTATCCTCCTAAAGTTACCTTCTAACGTCCATCCTAAAGCCAACACCTAAAGCCTACACCTAAAGACCCATCAAGTCAACGCCTCTCTTAAAGTTTAAACATAAAGACCACACCTAACGACCAGACCTAAAGACAATACATAAAGACAAAACCTAAAGACGCCTTGTTTTTa                                                                                                                                 >  1:162713/1‑301 (MQ=37)`  
                                                                     `tCACCTAAAGTCAACCTTCGGTTGACCTTGAGGGTT‑CCCTAAGGGTTGGGGATGACCCTTGGGTTTGTCTTTGGGTGTTACCTTGAGTGTCTCTCTGTGT‑CCCTATCTGTTACAGTCTCCTAAAGTATCCTCCTAAAGTCACCTCCTAACGTCCATCCTAAAGCCAACACCTAAAGCCTACACCTAAAGACCCATCAAGTCAACGCCTATCTTAAAGTTTAAACATAAAGACCAGACCTAAAGACCAGACCTAAAGACACTACATAAAGACCAGACCTAAAGACGCCTTGTTGTTAGCCAt                                                                                                                            >  1:680968/1‑301 (MQ=255)`  
                                                                        `ccTAAAGTCAACCTTCGGTTGACCTTGAGGGTT‑CCCTAAGGGTTGGGGATGACCCTTGGGTTTGTCTTTGGGTGTTACCTTGAGTGTCTCTCTGTGT‑CCCTATCTGTTCCAGTCTCCTAAAGTATCCT‑CTAAAGTCACCTCCTAACGTCCATCCTAAAGCCAACACCTAAAGCCTACACCTAAAGACCCATCAAGTCAACGCCTATCTTAAAGTTTAAACATAAAGACCAGACCTAAAGACCAGACCTAAAGACACTACATAAAGACCAGCCCTAAAGACGCCTTGTTGTTAGCCATaaaa                                                                                                                        >  1:206823/1‑300 (MQ=255)`  
                                                                              `gTCAACCTTCGGTTGACCTTGAGGGTT‑CCCTAAGGGTTGGGGATGACCCTTGGGTTTGTCTTTGGGTGTTACCTTGAGTGTCTCTCTGTGT‑CCCTATCTGTTACAGTCTCCTAAAGTATCCTCCTAAAGTCACCTCCTAACGTCCATCCTAAAGCCAACACCTAAAGCCTACACCTAAAGACCCATCAAGTCAACGCCTATCTTAAAGTTTAAACATAAAGACCAGACCTAAAGACCAGACCTAAAGACACTACATAAAGACCAGACCTAAAGACGCCTTGTTGTTAGCCATAAAGTGATa                                                                                                                   >  1:417405/1‑301 (MQ=255)`  
                                                                                 `tataagagacagACCTTGAGGGTT‑CCCTAAGGGTTGGGGATGACCCTTGGGTTTGTCTTTGGGTGTTACCTTGAGTGTCTCTCTGTGT‑CCCTATCTGTTCCAGTCTCCTAAAGTATCCTCCTAAAGTCACCTCCTAACGTCCATCCTAAAGCCAACACCTAAAGCCTACACCTAAAGACCCATCAAGTCAACGCCTATCTTAAAGTTTAAACATAAAGACCAGACCTAAAGACCAGACCTAAAGACACTACATAAAGACCAGACCTAAAGACGCCTTGTTGTTAGCCATAAAGTGATAAcc                                                                                                                <  1:234796/290‑1 (MQ=255)`  
                                                                                 `agacagcGGTTGACCTTGAGGGTT‑CCCTAAGGGTTGGGGATGACCCTTGGGTTTGTCTTTGGGTGTTACCTTGAGTGTCTCTCTGTGT‑CCCTATCTGTTACAGTCTCCTAAAGTATCCTCCTAAAGTCACCTCCTAACGTCCATCCTAAAGCCAACACCTAAAGCCTACACCTAAAGACCCATCAAGTCAACGCCTATCTTAAAGTTTAAACATAAAGACCAGACCTAAAGACCAGACCTAAAGACACTACATAAAGACCAGACCTAAAGACGCCTTGTTGTTAGCCATAAAGTGATAAcc                                                                                                                <  1:394980/295‑1 (MQ=255)`  
                                                                                  `aCCTTTGGTTGACCTTGAGGGTT‑CCCTAAGGGTTGGGGATGACCCTTGGGTTTGTCTTTAGGTGTTACCTTGAGTGTCTCTCTGTGT‑CCCGATCTGTTACAGTCTCCTAAAGTATCCTCCTAAAGTCACCTCCTAACGTCCATCCTAAAGCCAACACCTAAAGCCTACACCTAAAGACCCATCAAGTCAACGCCTATCTTAAAGTTTAAACATAAAGACCAGACCTAAAGACCAGACCTAAAGACACTACATAAAAACCAGACCTAAAGACGCCTTGTTGTTAGCCATAACGTGATAACCt                                                                                                               >  1:495610/1‑301 (MQ=255)`  
                                                                                      `tCGGTTGACCTTGAGGGTT‑CCCTAAGGGTTGGGGATGACCCTTGGGTTTGTCTTTGGGTGTTACCTTGTGTGTCTCTCTGTGT‑CCCTATCTGTTACAGTCTCCTAAAGTATCCTCCTAAAGTAACCTCCTAACGTCCATCCTAAAGCCAACACCTAAAGCCTACACCTAAAGACCCATCAAGTCAACGCCTATCTTAAAGTTTAAACATAAAGACCAGACCTAAAGACCAGACCTAAAGACACTACATAAAGACCAGACCTAAAGACGCCTTGTTGTTAGCCATCAAGTGATAACCTTTaa                                                                                                           >  1:688169/1‑301 (MQ=255)`  
                                                                                      `tCGGTTGACCTTGAGGGTT‑CCCTAAGGGTTGGGGATGACCCTTGGGTTTGTCTTTGGGTGTTACCTTGAGTGTCTCTCTGTGT‑CCCGATCTGTTACAGTCTCATAAAGTATCCTCCTAAAGTCACCTCCTAACGTCCATCCTAAAGCCAACACCTAAAGCCTACACCTAAAGACCCATCAAGTCAACGCCTATCTTAAAGTTTAAACATAAAGACCAGACCTAAAGACCAGACCTAAAGACACTACATAAAGACCAGACCTAAAGACGCCTTGTTGTTAGCCATAAAGTGATAACCTTTaa                                                                                                           >  1:56812/1‑301 (MQ=255)`  
                                                                                        `ggTTGACCTTGAGGGTT‑CCCTAAGGGTTGGGGATGACCCTTGGGTTTGTCTTTGGGTGTTACCTTGAGTGTCTCTCTGTGT‑CCCTATCTGTTACAGTCTCCTAAAGTATCCTCCTAAAGTCACCTCCTAACGTCCATCCTAAAGCCAACACCTAAAGCCTACACCTAAAGACCCATCAAGTCAACGCCTATCTTAAAGTTTAAACATAAAGACCAGACCTAAAGACCAGACCTAAAGACACTACATAAAGACCAGACCTAAAGACGCCTTGTTGTTAGCCATAAAGTAATAACCTTTAATc                                                                                                         <  1:803973/301‑1 (MQ=255)`  
                                                                                        `ggTTGACCTTGAGGGTT‑CCCTAAGGGTTGGGGATGACCCTTGGGTTTGTCTTTGGGTGTTACCTTGAGTGTCTCTCTGTGT‑CCCGATCTGTTACAGTCTCCTAAAGTATCCTCCTAAAGTCACCTCCTAACGTCCATCCTAAAGCCAACACCTAAAGCCTACACCTAAAGACCCATCAAGTCAACGCCTATCTTAAAGTTTAAACATAAAGACCAGACCTAAAGACCAGACCTAAAGACACTACATAAAGACCAGACCTAAAGACGCCTTGTTGTTAGCCATAAAGTGATAACCTTTAATc                                                                                                         <  1:439568/301‑1 (MQ=255)`  
                                                                                         `gacagccTTGAGGGTT‑CCCTAAGGGTTGGGGATGACCCTTGGGTTTGTCTTTGGGTGTTACCTTGAGTGTCTCTCTGTGT‑CCCGATCTGTTACAGTCTCCTAAAGTATCCTCCTAAAGTCACCTCCTAACGTCCATCCTAAAGCCAACACCTAAAGCCTACACCTAAAGACCCATCAAGTCAACGCCTATCTTAAAGTTTAAACATAAAGACCAGACCTAAAGACCAGACCTAAAGACACTACATAAAGACCAGACCTAAAGACGCCTTGTTGTTAGCCATAAAGTGATAACCTTTAATCa                                                                                                        <  1:651408/296‑1 (MQ=255)`  
                                                                                         `gTTGACCTTGAGGGTT‑CCCTAAGGGTTGGGGATGACGCTTGGGTTTGTCTTTGGGTGTTACCTTGAGTGTCTCTCTGTGT‑CCCTATCTGTTACAGTCTCCTAAAGTATCCTACTAAAGTCACCTCCTAACGTCCATCCTAAAGCCAACACCTAAAGCCTACACCTAAAGACCCATCAAGTCAACGCCTATCTTAAAGTTTAAACATAAAGACCAGACCTAAAGACCAGACCTAAAGACACTACATAAAGACCAGACCTAAAGACGCCTTGTTGTTAGCCATAAAGTGATAACCTTTAATCa                                                                                                        <  1:767633/301‑1 (MQ=255)`  
                                                                                            `gACCTTGAGGGTT‑CCCTAAGGGTTGGGGATGACCCTTGGGTTTGTCTTTGGGTGTTACCTTGAGTGTCTCTCTGTGT‑CCCTATCTGTTACAGTCTCCTAAAGTATCCTCCTAAAGTCACCTCCTAACGTCCATCCTAAAGCCAACACCTAAAGCCTACACCTAAAGACCCATCAAGTCAACGCCTATCTTAAAGTTTAAACATAAAGACCAGACCTAAAGACCAGACCTAAAGAAACTACATAAAGACAAGACCTAAAGACGCCTTGTTGTTAGCCATAAAGTGATAACCTTTAATCAttt                                                                                                     >  1:77648/1‑300 (MQ=255)`  
                                                                                            `gACCTTGAGGGTT‑CCCTAAGGGTTGGGGATGACCCTTGGGTTTGTCTTTGGGTGTTACCTTGAGTGTCTCTCTGTGT‑CCCTATCTGTTACAGTCTCCTAAAGTATCCTCCTAAAGTCACCTCCTAACGTCCATCCTAAAGCCAACACCTAAAGCCTACACCTAAAGACCCATCAAGTCAACGCCTATATTAAAGTTTAAACATAAAGACCAGACCTAAAGACCAGACCTAAAGACACTACATAAAGACCAGACCTAAAGACGCCTTGTTGTTAGCCATAAAGTGATAACCTTTAATcctgt                                                                                                     >  1:303894/1‑297 (MQ=255)`  
                                                                                              `ccTTGAGGGTT‑CCCTAAGGGTTGGGGATGACCCTTGGGTTTGTCTTTGGGTGTTACCTTGAGTGTCTCTCTGTGT‑CCCTATCTGTTACAGTCTCCTAAAGTATCCTCCTAAAGTCACCTCCTAACGTCCATCCTAAAGCCAACACCTAAAGCCTACACCTAAAGACCCATCAAGTCAACGCCTATCTTAAAGTTTAAACATAAAGACCAGACCTAAAGACCAGACCTAAAGACACTACATAAAGACCAGACCTAAAGACGCCTTGTTGTTAGCCATAAAGTGATAACCTTTAATcctgtct                                                                                                   >  1:560551/1‑295 (MQ=255)`  
                                                                                                `taagagacagcccTAAGGGTTGGGGATGACCCTTGGGTTTGTCTTTGGGTGTTACCTTGAGTGTCTCTCTGTGT‑CCCTATCTGTTCCAGTCTCCTAAAGTATCCTCCTAAAGTCACCTCCTAACGTCCATCCTAAAGCCAACACCTAAAGCCTACACCTAAAGACCCATCAAGTCAACGCCTATCTTAAAGTTTAAACATAAAGACCAGACCTAAAGACCAGACCTAAAGACACTACATAAAGACCAGACCTAAAGACGCCTTGTTGTTAGCCATAAAGTGATAACCTTTAATCATTGTCt                                                                                                  <  1:169859/291‑1 (MQ=255)`  
                                                                                                    `gggTT‑CCCTAAGGGTTGGTGATGACCCGTGGGTTTGTCTTTGGGTGTTACCTTGAGTGTCTCTCTGTGT‑CCCTATCTGTTACAGTCTCCTAAAGTATCCTCCTAAAGTCACCTCCTAACGTCCATCCTAAAGCCAACACCTAAAGCCTACACCTAAAGACCCATCAAGTCAACGCCTATCTTAAAGTTTAAACATAAAGACCAGACCTAAAGACCAGACCTAAAGACACTACATAAAGACCAGACCTAAAGACGCCTTGTTGTTAGCCATAAAGTGATAACCTTTAATCATTGTCTttatt                                                                                             <  1:100228/301‑1 (MQ=255)`  
                                                                                                          `cccTAAGGGTTGGGGATGACCCTTGGGTTTGTCTTTGGGTGTTACCTTGAGTGTCTCTCTGTGT‑CCCTATCTGTTACAGTCTCCTAAAGTATCCTCCTAAAGTCACCTCCTAACGTCCATCCTAAAGCCAACACCTAGAGCCTACACCTAAAGACCCATCAAGTCAACGCCTATCTTAAAGTTTAAACATAAAGACCAGACCTAAAGACCAGACCTAAAGACACTACATAAAGACCAGACCTAAAGACGCCTTGTTGTTAGCCATAAAGTGATAACCTTTAATCATTGTCTTTATTAATAc                                                                                        >  1:32973/1‑301 (MQ=255)`  
                                                                                                          `cccTAAGGGTTGGGGATGACCCTTGGGTTTGTCTTTGGGTGTTACCTTGAGTGTCTCTCTGTGT‑CCCTATCTGTTACAGTCTCCTAAAGTATCCTCCTAAAGTCACCTCCTAACGTCCATCCTAAAGCCAACACCTAAAGCCTACACCTAAAGACCCATCAAGTCAACGCCTATCTTAAAGTTTAAACATAAAGACCAGACCTAAAGACCAGACCTAAAGACACTACATAAAGACCAGACCTAAAGACGCCTTGTTGTTAGCCATAAAGTGATAACCTTTAATAATTGTCTTTATTAATAc                                                                                        <  1:25638/301‑1 (MQ=255)`  
                                                                                                           `ccTAAGGGTTGGGGATGACCCTTGGGTTTGTCTTTGGGTGTTACCTTGAGTGTCTCTCTGTGTCCCCTATCTGTTACAGTCTCCTAAAGTATCCTCCTAAAGTCACCTCCTAACGTCCATCCTAAAGCCAACACCTAAAGCCTACACCTAAAGACCCATCAAGTCAACGCCTATCTTAAAGTTTAAACATAAAGACCAGACCTAAAGACCAGACCTAAAGACACTACATAAAGACCAGACCTAAAGACGCCTTGTTGTTAGCCATAAAGTGATAACCTTTAATCATTGTCTTTATTAATAc                                                                                        >  1:659034/1‑301 (MQ=255)`  
                                                                                                               `aGGGTTGGGGATGACCCTTGGGTTTGTCTTTGGGTGTTACCTTGAGTGTCTCTCTGTGT‑CCCTATCTGTTACAGTCTCCTAAAGTATCCTCCTAAAGTCACCTCCTAACGTCCATCCTAAAGCCAACACCTAAAGCCTACACCTAAAGACCCATCAAGTCAACGCCTATCTTAAAGTTTAAACATAAAGACCAGACCTAAAGACCAGACCTAAAGACACTACATAAAGACCAGACCTAAAGACGCCTTGTTGTTAGCCATAAAGTGATAACCTTTAATCATTGTCTTTATTAATACAACTc                                                                                   <  1:26336/301‑1 (MQ=255)`  
                                                                                                               `aGGGTTGGGGATGACCCTTGGGTTTGTCTTTGGGTGTTACCTTGAGTGTCTCTCTGTGT‑CCCTATCTGTTACAGTCTCCTAAAGTATCCTCCTAAAGTCACCTCCTAACGTCAATACTAAAGCCAACACCTAAAGCCTACACCTAAAGACCCATCAAGTCAACGCCTATCTTAAAGTTTAAACATAAAGACCAGACCTAAAGACCAGACCTAAAGACACTACATAAAGACCAGACCTAAAGACGCCTTGTTGTTAGCCATAAAGTGATAACCTTTAATCATTGTCTTTATTAATACAACTc                                                                                   <  1:316642/301‑1 (MQ=255)`  
                                                                                                               `aGGGTTGGGGAGGACCCTTTGGTTTGTCTTTGGGTGTTACCTTGAGTGTCTCTCTGTGT‑CCCTATCTGTTACAGTCTCCTAAAGTATCCTCCTAAAGTCACCTCCTAACGTCCATCCTAAAGCCAACACCTAAAGCCTACACCTAAAGACCCATCAAGTCAACGCCTATCTTAAAGTTTAAACATAAAGACCAGACCTAAAGACCAGACCTAAAGACACTACATAAAGACCAGACCTAAAGACGCCTTGTTGTTAGCCATAAAGTGATAACCTTTAATCATTGTCTTTATTAATACAACTc                                                                                   <  1:758802/301‑1 (MQ=255)`  
                                                                                                                `gggTTGGGGATGACCCTTGGGTTTGTCTTTGGGTGTTACCTTGAGTGTCTCTCTGTGT‑CCCTATCTGTTACAGTCTCCTAAAGTATCCTCCTAAAGTCACCTCCTAACGTCCATCCTAAAGCCAACACCTAAAGCCTACACCTAAAGACCCATCAAGTCAACGCCTATCTTAAAGTTTAAACATAAAGACCAGACCTAAAGACCAGACCTAAATACACTACATAAAGACCAGACCTAAAGACGCCTTGTTGTTAGCCATAAAGTGATAACCTTTAATCATTGTCTTTATTAATACAACTCa                                                                                  >  1:725474/1‑301 (MQ=255)`  
                                                                                                                `gggTTGGGGATGACCCTTGGGTTTGTCTTTGGGTGTTACCTTGAGTGTCTCTCTGTGT‑CCCTATCTGTTACAGTCTCCTAAAGTATCCTCCTAAAGTCACCTCCTAACGTCCATCCTAAAGCCAACACCTAAAGCCTACACCTAAAGACCCATCAAGTCAACGCCTATCTTAAAGTTTAAACATAAAGACCAGACCTAAAGACCAGACCTAAAGACACTACATAAAGACCAAACCTAAAGACGCCTTGTTGTTAGCCATAAAGTGATAACCTTTAATCATTGTCTTTATTAATACAACTCa                                                                                  >  1:446618/1‑301 (MQ=255)`  
                                                                                                                      `gggATGACCCTTGGGTTTGTCTTTGGGTGTTACCTTGAGTGGCTCTCTGTGT‑CCCTATCTGTTCCAGTATCCTAAAGTATCCTCCTAAAGTCACCTCCTAACGTCCAGCCTAAAGCCAACACCTAAAGCCTACACCTAAAGACCCATCAAGTCAACGCCTATCTTAAAGTTTAAACATAAAGACCAGACCTAAAGACCAGACCTAAAGACACTACATAAAGACCAGACCTAAAGACGCCTTGTTGTTAGCCATAAAGTGATAACCTTTAATCATTGTCTTTATTAATACAACTCACTATaa                                                                            <  1:298641/301‑1 (MQ=255)`  
                                                                                                                       `ggATGACCCTTGGGTTTGTCTTTGGGTGTTACCTTGAGTGTCTCTCTGTGT‑CCCGATCTGTTACAGTCTCCTAAAGTATCCTCCTAAAGTCACCTCCTAACGTCCATCCTAAAGCCAACACCTAAAGCCTACACCTAAAGACCCATCAAGTCAACGCCTATCTTAAAGTTTAAACATAAAGACCAGACCTAAAGACCAGACCTAAAGACACTACATAAAGACCAGACCTAAAGACGCCTTGTTGTTAGCCATAAAGTGATAACCTTTAATCATTGTCTTTATTAATACAACTCACTATAAg                                                                           <  1:636564/301‑1 (MQ=255)`  
                                                                                                                        `gATGACCCTTGGGTTTGTCTTTGGGTGTTACCTTGAGTGTCTCTCTGTGT‑CCCGATCTGTTACAGTCTCCTAAAGTATCCTCCTAAAGTCACCTCCTAACGTCCATCCTAAAGCCAACACCTAAAGCCTACACCTAAAGACCCATCAAGTCAACGCCTATCTTAAAGTTTAAACATAAAGACCAGACCTAAAGACCAGACCTAAAGACACTACATAAAGACCAGACCTAAAGACGCCTTGTTGTTAGCCATAAAGTGATAACCTTTAATCATTGTCTTTATTAATACAACTCACTATAAgg                                                                          <  1:508575/301‑1 (MQ=255)`  
                                                                                                                        `gATGAACCTTGGGTTTGTCTTTGGGTGTTACCTTGAGTGTCTCTCTGTGT‑CCCTATCTGTTACAGTCTCCTAAAGTATCCTCCTAAAGTCACCTCCTAACGTCCATCCTAAAGCCAACACCTAAAGCCTACACCTAAAGACCCATCAAGTCAACGCCTATCTTAAAGTTTAAACATAAAGACCAGACCTAAAGACCAGACCTAAAGACACTACATAAAGACCAGACCTAAAGACGCCTTGTTGTTAGCCATAAAGTGATAACCTTTAATCATTGTCTTTATTAATACAACTCACTATAAgg                                                                          <  1:664305/301‑1 (MQ=255)`  
                                                                                                                         `aggACCCTTGGGTTTGTCTTTGGGTGTTACCTTGAGTGTCTCTCTGTGT‑CCCTATCTGTTCCAGTCGCCTAAAGTATCCTCCTAAAGTCACCTCCTAACGTCCATCCTAAAGCCAACACCTAAAGCCTACACCTAAAGACCCATCAAGTCAACGCCTATCTTAAAGTTTAAACATAAAGACCAGACCTAAAGACCAGACCTAAAGACACTACATAAAGACCAGACCTAAAGACGCCTTGTTGTTAGCCATAAAGTGATAACCTTTAATCATTGTCTTTATTAATACAACTCACTATAAGga                                                                         <  1:54435/299‑1 (MQ=255)`  
                                                                                                                         `aTGACCCTTGGGTTTGTCTTTGGGTGTTACCTTGAGTGTCTCTCTGTGT‑CCCTATCTGTTACAGTCTCCTAAAGTATCCTCCTAAAGTCACCTCCTAACGTCCATCCTAAAGCCAACACCTAAAGCCTACATCTAAAGACCCATCAAGTCAACGCCTATCTTAAAGTTTAAACATAAAGACCAGACCTAAAGACCAGACCTAAAGACACTACATAAAGACCAGACCTAAAGACGCCTTGTTGTTAGCCATAAAGTGATAACCTTTAATCATTGTCTTTATTAATACAACTCACTATAAGga                                                                         <  1:606328/301‑1 (MQ=255)`  
                                                                                                                          `tGACCCTTGTGTTTGTCTTTGGGTGTTACCTTGAGTGTCTCTCTGTGT‑CCCTATCTGTTACAGTCTCCTAAAGTATCCTCCTAAAGTCACCTCCTAACGTCCATCCTAAAGCCAACACCTAAAGCCTACACCTAAAGACCCATCAAGTCAACGCCTATCTTAAAGTTTAAACATAAAGACCAGACCTAAAGACCAGACCTAAAGACACTACATAAAGACCAGACCTAAAGACGCCTTGTTGTTAGCCATAAAGTGATAACCTTTAATCATTGTCTTTATTAATACAACTCACGATAAGgag                                                                        <  1:672933/301‑1 (MQ=255)`  
                                                                                                                             `cccTTGGGTTTGTCTTTGGGTGTTACCTTGAGTGTCTCTCTGTGT‑CCCTATCTGTTACAGTCTCCTAAAGTATCCTCCTAAAGTCACCTCCTAACGTCCATCCTAAAGCCAACACCTAAAGCCTACACCTAAAGACCCATCAAGTCAACGCCTATCTTAAAGTTTAAACATAAAGACCAGACCTAAAGACCAGACCTAAAGACACTACATAAAGACCAGACCTAAAGACGCCTTGTTGTTAGCCATAAAGTGATAACCTTTAATCATTGTCTTTATTAATACAACTCACtatctgtctctt                                                                     >  1:271476/1‑292 (MQ=255)`  
                                                                                                                             `cccTTGGGTTTGTCTTTGGGTGTTACCTTGAGTGTCTCTCTGTGT‑CCCTATCTGTTACAGTCTCCTAAAGTATCCTCCTAAAGTCACCTCCTAACGTCCATCCTAAAGCCAACACCTAAAGCCTACACCTAAAGACCCATCAAGTCAACGCCTATCTTAAAGTTTAAACATAAAGACCAGACCTAAAGACCAGACCTAAAGACACTACATAAAGACCAGACCTAAAGACGCCTTGTTGTTAGCCATAAAGTGATAACCTTTAATCATTGTCTTTATTAATACAACTCACTATAAGgagaga                                                                     >  1:226971/1‑301 (MQ=255)`  
                                                                                                                               `cTTGGGTTTGTCTTTGGGTGTTACCTTGAGTGTCTCTCTGTGT‑CCCTATCTGTTACAGTCTCCTAAAGTATCCTCCTAAAGTCACCTCCTAACGTCCATCCTAAAGCCAACACCTAAAGCCTACACCTAAAGACCCATCAAGTCAACGCCTATCTTAAAGTTTAAACATAAAGACCAGACCTAAAGACCAGACCTAAAGACACTACATAAAGACCAGACCTAAAGACGCCTTGTTGTTAGCCATAAAGTGATAACCTTTAATCATTGTCTTTATTAATACAACTAACTATAAGGAGAGACa                                                                   <  1:358931/301‑1 (MQ=255)`  
                                                                                                                                  `gggTTTGTCTTTGGGTGTTACCTTGAGTGTCTCTCTGTGT‑CCCTATCTGTTACAGTCTCCTAAAGTATCCTCCTAAAGTCACCTCCTAACGTCCATCCTAAAGCCAACACCTAAAGCCTACACCTAAAGACCCATCAAGTCAACGCCTATCTTAAAGTTTAAACATAAAGACCAGCCCTAAAGACCAGACCTAAAGACACTACATAAAGACCAGACCTAAAGACGCCTTGTTGTTAGCCATAAAGTGATAACCTTTAATCATTGTCTTTATTAATACAACTCACTATAAGGAGAGACAACt                                                                >  1:220612/1‑301 (MQ=255)`  
                                                                                                                                       `gtagaagagacagtACCTTTAGTGTCTCTCTGTGT‑CCCTATCTGTTACAGTCTCCTAAAGTATCCTCCTAAAGTCACCTCCTAACGTCCATCCTAAAGCCAACACCTAAAGCCTACACCTAAAGACCCATCAAGTCAACGCCTATCTTAAAGTTTAAACATAAAGACCAGACCTAAAGACCAGACCTAAAGACACTACATAAAGACCAGACCTAAAGACGCCTTGTTGTTAGCCATAAAGTGATAACCTTTAATCATTGTCTTTATTAATACAACTCACTATAAGGAGAGACAACTTAAag                                                           <  1:387086/288‑1 (MQ=255)`  
                                                                                                                                       `aagagacaggTGTTACCTTGAGTGTCTCTCTGTGT‑CCCTATCTGTTACAGTCTCCTAAAGTATCCTCCTAAAGTCACCTCCTAACGTCCATCCTAAAGCCAACACCTAAAGCCTACACATAAAGACCCATCAAGTCAACGCCTATCTTAAAGTTTAAACATAAAGACCAGACCTAAAGACCAGACCTAAAGACACTACATAAAGACCAGACCTAAAGACGCCTTGTTGTTAGCCATAAAGTGATAACCTTTAATCATTGTCTTTATTAATACAACTCACTATAAGGAGAGACAACTTAAag                                                           <  1:600215/293‑1 (MQ=255)`  
                                                                                                                                        `gTCTTTGGGTGTTACCTTGAGTGTCTCTCTGTGTCCCCTATCTGTTACAGTCTCCTAAAGTATCCTCCTAAAGTCACCTCCTAACGTCCATCCTAAAGCCAACACCTAAAGCCTACACCTAAAGACCCATCAAGTCAACGCCTATCTTAAAGTTTAAACATAAAGACCAGACCTAAAGACCAGACCTAAAGACACTACATAAAGACCAGACCTAAAGACGCCTTGTTGTTAGCCATAAAGTGATAACCTTTAATCATTGTCTTTATTAATACAACTCACTATAAggctgtctcttatacac                                                           >  1:519534/1‑286 (MQ=255)`  
                                                                                                                                        `gTCTTTGGGTGTTACCTTGAGTGTCTCTCTGTGT‑CCCTATCTGTTACAGTCTCCTAAAGTATCCTCCTAAAGTCACCTCCTAACGTCCATCCTAAAGCCAACACCTAAAGCCTACACCTAAAGACCCATCAAGTCAACGCCTATCTTAAAGTTTAAACATAAAGACCAGACCTAAAGACCAGACCTAAAGACACTACATAAAGACCAGACCTAAAGACGCCTTGTTGTTAGCCATAAAGTGATAACCTTTAATCATTGTCTTTATTAATAcctgtctcttatacacatctccgagcccacc                                                          >  1:454025/1‑271 (MQ=255)`  
                                                                                                                                        `gTCTTTGGGTGTTACCTTGAGTGTCTCTCTGTGT‑CCCTATCTGTTACAGTCTCCTAAAGTATCCTCCTAAAGTCACCTCCTAACGTCCATCCTAAAGCCAACACCTAAAGCCTACACCTAAAGACCCATCAAGTCAACGCCTATCTTAAAGTTTAAACATAAAGACCAGACCTAAAGACCAGACCTAAAGACACTACATAAAGACCAGACCTAAAGACGCCTTGTTGTTAGCCATAAAGTGATAACCTTTAATCATTGTCTTTATTAATACAACTCACTATAAGGAGAGACAACTTAAaga                                                          >  1:364876/1‑301 (MQ=255)`  
                                                                                                                                        `gTCTTTGGGTGTTACCTTGAGTGTCTCTCTGTGT‑CCCTATCTGTTACAGTCTCCTAAAGTATCCTCCTAAAGTCACCTCCTAACGTCCATCCTAAAGCCAACACCTAAAGCCTACACCTAAAGACCCATCAAGTCAACGCCTATCTTAAAGTTTAAACATAAAGACCAGACCTAAAGACCAGACCTAAAGACACTACATAAAGACCAGACCTAAAGACGCCTTGTTGTTAGCCATAAAGTGATAACCTTTAATCATTGTCTTTATTAATACAACTCACTATAAGGAGAGACAACTTAAaga                                                          >  1:137877/1‑301 (MQ=255)`  
                                                                                                                                              `gggTGTTACCTTGAGTGTCTCTCTGTGT‑CCCGATCTGTTACAGTCTCCTAAAGTATCCTCCTAAAGTCACCTCCTAACGTCCATCCTAAAGCCAACACCTAAAGCCTACACCTAAAGACCCATCAAGTCAACGCCTATCTTAAAGTTTAAACATAAAGACCAGACCTAAAGACCAGACCTAAAGACACTACATAAAGACCAGACCTAAAGACGCCTTGTTGTTAGCCATAAAGTGATAACCTTTAATCATTGTCTTTATTAATACAACTCACTATAAGGAGCGACAACTTAAAGAGACTTa                                                    >  1:703897/1‑301 (MQ=255)`  
                                                                                                                                               `ggTGTTACCTTGAGTGTCTCTCTGTGT‑CCCTATCTGTTACAGTCTCCTAAAGTATCCTCCTAAAGTCACCTCCTAACGTCCATCCTAAAGCCAACACCTAAAGCCTACACCTAAAGACCCATCAAGTCAACGCCTATCTTAAAGTTTAAACATAAAGACCAGACCTAAAGACCAGACCTAAAGACACTACATAAAGACCAGACCTAAAGACGCCTTGTTGTTAGCCATAAAGTGATAACCTTTAATCATTGTCTTTATTAATACAACTCACTATAAGGAGAGACAACTTAAAGAGACTTaa                                                   >  1:711185/1‑301 (MQ=255)`  
                                                                                                                                                `gtgtTACCTTGAGTGTCTCTCTGTGT‑CCCTATCTGTTACAGTCTCCTAAAGTATCCTCCTAAAGTCACCTCCTAACGTCCATCCTAAAGCCAACACCTAAAGCCTACACCTAAAGACCCATCAAGTCAACGCCTATCTTAAAGTTTAAACATAAAGACCAGACCTAAAGACCAGACCTAAAGACACTACATAAAGACCAGACCTAAAGACGCCTTGTTGTTAGCCATAAAGTGATAACCTTTAATCATTGTCTTTATTAATACAACTCACTATAAGGAGAGACAACTTAAagctgtctctt                                                  >  1:9361/1‑292 (MQ=255)`  
                                                                                                                                                    `tACCTTGAGTGTCTCTCTGTGT‑CCCTATCTGTTACAGTCTCCTAAAGTATCCTCCTAAAGTCACCTCCTAACGTCCATCCTAAAGCCAACACCTAAAGCCTACACCTAAAGACCCATCAAGTCAACGCCTATCTTAAAGTTTAAACATAAAGACCAGACCTAAAGACCAGACCTAAAGACACTACATAAAGACCAGACCTAAAGACGCCTTGTTGTTAGCCATAAAGTGATAACCTTTAATCATTGTCTTTATTAATACAACTCACTATAAGAATAGCCAACTTAAAGAGACTTAAAAGAt                                              >  1:115004/1‑301 (MQ=255)`  
                                                                                                                                                    `tACCTTGAGTGTCTCTCTGTGT‑CCCGATCTGTTACAGTCTCCTAAAGTATCCTCCTAAAGTCACCTCCTAACGTCCATCCTAAAGCCAACACCTAAAGCCTACACCTAAAGACCCATCAAGTCAACGCCTATCTTAAAGTTTAAACATAAAGACCAGACCTAAAGACCAGACCTAAAGACACTACATAAAGACCAGACCTAAAGACGCCTTGTTGTTAGCCATAAAGTGATAACCTTTAATCATTGTCTTTATTAATACAACTCACTATAAGGAGAGACAACTTAAAGAGACTTAAAAGAt                                              >  1:467191/1‑301 (MQ=255)`  
                                                                                                                                                          `tataagagacaggtgt‑TCCTATCTGTTCCAGTCTCCTAAAGTATCCTCCTAAAGTCACCTCCTAACGTCCATCCTAAAGCCAACACCTAAAGCATACACCTAAAGACCCATCAAGTCAACGCCTATCTTAAAGTTTAAACATAAAGACCAGACCTAAAGACCAGACCTAAAGACACTACATAAAGACCAGACCTAAAGACGCCTTGTTGTTAGCCATAAAGTGATAACCTTTAATCATTGTCTTTATTAATACAACTCACTATAAGGAGAGACAACTTAAAGAGACTTAAAAGATTAAttt                                        <  1:792578/289‑1 (MQ=255)`  
                                                                                                                                                                           `cccTATCTGTTACAGTCTCCTAAAGTATCCTCCTAAAGTCACCTCCTAACGTCCATCCTAAAGCCAACACCTAAAGCCTACACCTAAAGACCCATCAAGTCAACGCCTATCTTAAAGTTTAAACATAAAGACCAGACCTAAAGACCAGACCTAAAGACACTACATAAAGACCAGACCTAAAGACGCCTTGTTGTTAGCCATAAAGTGATAACCTTTAATCATTGTCTTTATTAATACAACTCACTATAAGGAGAGACAACTTAAAGAGACTTAAAAGATTAATTTAAAATTTATCaaaaaa                        >  1:214127/1‑300 (MQ=255)`  

                                                                                                                                                                              `|`                                                                                                                                                                                                                                                                                                                                 
`‑‑‑‑‑‑‑‑‑‑‑‑‑TCTCACAGTGTACGGACCTAAAGTTCCCCCATAGGGGGTACCTAAAGCCCAGCCAATCACCTAAAGTCAACCTTCGGTTGACCTTGAGGGTT‑CCCTAAGGGTTGGGGATGACCCTTGGGTTTGTCTTTGGGTGTTACCTTGAGTGTCTCTCTGTGT‑CCCTATCTGTTACAGTCTCCTAAAGTATCCTCCTAAAGTCACCTCCTAACGTCCATCCTAAAGCCAACACCTAAAGCCTACACCTAAAGACCCATCAAGTCAACGCCTATCTTAAAGTTTAAACATAAAGACCAGACCTAAAGACCAGACCTAAAGACACTACATAAAGACCAGACCTAAAGACGCCTTGTTGTTAGCCATAAAGTGATAACCTTTAATCATTGTCTTTATTAATACAACTCACTATAAGGAGAGACAACTTAAAGAGACTTAAAAGATTAATTTAAAATTTATCAAAAAGAGTATTGACTTAAAGTCTAACC  >  NC_001604.1/1‑479`

  

Alignment Legend

Aligned base mismatch/match (shaded by quality score): `ATCG/ATCG` `< 3 ≤` `ATCG/ATCG` `< 15 ≤` `ATCG/ATCG` `< 27 ≤` `ATCG/ATCG` `< 37 ≤` `ATCG/ATCG` `< 38 ≤` `ATCG/ATCG`

Unaligned base: `atcg`    Masked matching base: `atcg`    Alignment gap: `‑`    Deleted base: `‑`
